# Gall responses to drying habitats: Insights from the community of galling herbivores associated with the superhost *Caryocar brasiliense* Cambess. (Caryocaraceae)

**DOI:** 10.1111/plb.70008

**Published:** 2025-04-04

**Authors:** I. S. Fernandes, W. S. Araújo, R. G. S. da Carneiro

**Affiliations:** ^1^ Programa de Pós‐Graduação em Botânica Aplicada Universidade Estadual de Montes Claros Montes Claros Minas Gerais Brazil; ^2^ Departamento de Biologia Geral Universidade Estadual de Montes Claros Montes Claros Minas Gerais Brazil; ^3^ Departamento de Botânica Instituto de Ciências Biológicas, Universidade Federal de Goiás Goiânia Goiás Brazil

**Keywords:** gall morphospecies, hymenopteran galls, interspecific competition, stress markers

## Abstract

- Galls are new plant organs formed in response to the stimuli of gall‐inducing organisms, but may also be influenced by environmental conditions.
- This study evaluated the richness, abundance, and co‐occurrence in the gall community associated with *Caryocar brasiliense* Cambess. (Caryocaraceae) areas with varying degrees of environmental stress due to the drying of palm swamps, called *Veredas*. Additionally, structural and metabolic attributes related to nutrition and protection were evaluated as possible stress markers. The research was conducted in two Cerrado areas with different water availability: the *Parque Estadual Veredas do Peruaçu* (lower water availability; more stressful) and the *Área de Proteção Ambiental do Rio Pandeiros* (higher water availability; less stressful).
- A total of 51,336 galls from five morphospecies were sampled: intralaminar globoid gall (IGG), extralaminar globoid gall (EGG), globoid petiolar gall (GPG), lenticular gall (LG) and clavate gall (CG). Gall richness was similar in both environments, but abundance was higher in the less stressful area, challenging the hypothesis that environmental stress increases gall formation. Random co‐occurrence patterns suggested that gall inducers occupy distinct spatial niches to reduce competition. Structurally and metabolically, galls exhibited similarities, including nutritive tissues and nutritious substances in their internal compartments. However, in the more stressful environment, some galls had smaller internal and larger external compartments, with increased lignin and phenolic compounds in the outer tissue layers.
- Lignin deposition and phenolic accumulation are evidenced as stress markers which indicate that galls display phenotypic plasticity and adaptive strategies, enhancing protection and survival under the stressful conditions of drying environments.

## Graphical abstract

Drying processes in the Brazilian palm swamps or *Veredas*, impact the gall community in adjacent vegetation by reducing the abundance of more sensitive galls, while more resilient galls acclimate to increased dryness by developing thicker protective tissues.

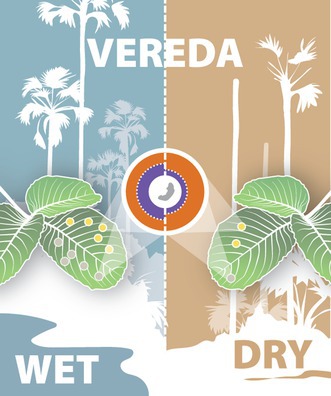

## INTRODUCTION

Galls are atypical organs formed by the structural and functional reconfiguration of host plant tissues, induced by various organisms (Mani 1964; Ferreira *et al*. 2019), but insect‐induced galls are the most common and complex, both structurally and metabolically (Isaias *et al*. 2014). The relationship between galling insects and host plants is species‐specific (Mani 1964), making each system unique, as gall development is directed by the inducer (Stone & Schönrogge 2003). Galls provide shelter, nutrition, and protection for their inducers (Price *et al*. 1987) via structural and chemical changes in plant tissues that affect metabolite production, cell structure (Oliveira *et al*. 2006), and tissue growth, leading to the formation of diverse gall morphotypes (Isaias *et al*. 2013).

Various factors explain patterns in galling insect communities and gall formation. The environmental stress hypothesis (Fernandes & Price 1988) suggests higher gall richness in harsh habitats with limited water and nutrients. Some studies confirmed this pattern, showing greater gall richness and lower mortality rates of gall inducers in xeric compared to mesic environments (Fernandes & Price 1992; Gonçalves‐Alvim & Fernandes 2001), while other studies refute this hypothesis (Cuevas‐Reyes *et al*. 2003; Arriola *et al*. 2015). Interspecific competition also plays a role, as competing species rarely coexist without resource differentiation (Begon *et al*. 2007). Evidence suggests that competition influences gall community structure through the selection of induction sites and regulation of gall development (Fagundes *et al*. 2020), allowing different taxa to occupy the same plant organs without direct competition. Hence, the anatomical and histochemical diversity in galls results from the recruitment of different cellular and metabolic mechanisms for gall maintenance (Ferreira *et al*. 2019), which explains how distinct structural traits of galls are found on a single galled organ. Structural differences, including anatomical traits, are key to distinguishing gall morphotypes and morphospecies even when inducers are not identified (Teixeira *et al*. 2022).

*Caryocar brasiliense* Cambess. (Caryocaraceae) is a Brazilian Cerrado species with significant socioeconomic importance for traditional communities (Araújo 1995; Almeida *et al*. 1998). It also has an important ecological role due to its diverse associated entomofauna, especially galling insects. Various insect gall morphotypes are associated with the leaves of *C. brasiliense* (Urso‐Guimarães *et al*. 2003; Castro *et al*. 2012; Leite 2014), making it a ‘superhost’ of galling insects (Isaias *et al*. 2013). Among the gall morphotypes on *C. brasiliense*, four are induced by Hymenoptera species (Leite 2014), with shapes such as globoid, lenticular, and clavate (see Material and Methods and Results). These galls, induced by different species, are designated as ‘morphospecies’ (*sensu* Santana & Isaias 2014; Teixeira *et al*. 2022), referring to specific gall shapes induced by a particular galling species, rather than just a ‘morphotype,’ which only describes the shape.

As a widespread plant species in the Brazilian Cerrado, *C. brasiliense* grows in nutrient‐poor soils and has a deep, pivoting root system. However, 80% of its roots are at depths around 1.25 m (Lima *et al*. 2015), making it susceptible to water deficits in superficial soil layers. Despite the adaptations to harsh Cerrado environments, such as dense indumentum and compact mesophyll on leaves (Ramos *et al*. 2015), little is known about how environmental changes affect these plants and their associated galling fauna amid increasing dryness due to habitat loss and climate changes. Although functional anatomical attributes are well known and discussed in terms of plant adaptability (De Micco & Aronne 2012; Pérez‐Harguindeguy *et al*. 2013), they are yet to be described and standardized for galls. Nevertheless, anatomical traits such as dense indumentum and thick cuticle in the epidermis, and increased thickness, compactness and sclerophylly in ground tissues (Isaias *et al*. 2014, 2024; Ferreira *et al*. 2019), as well as the accumulation of secondary metabolites, such as alkaloids, terpenoids and phenolics in the outermost tissue layers, help galls to withstand stressful environments (Kuster *et al*. 2020). This is particularly relevant in northern Minas Gerais, Brazil, where palm swamps (*Veredas*) are drying out due to anthropogenic activities, leading to groundwater level retraction and increased local dryness. This drying process has caused a loss of typical floristic composition of the drying *Veredas*, where plants that depend on high soil humidity are gradually dying (Nunes *et al*. 2022). Although *C. brasiliense* and its galls are not typically found within the *Veredas*, they occur adjacent to these areas, making them excellent models to study how typical Cerrado plants and fauna respond to environmental changes and drying processes, particularly given recent anthropogenic threats to Cerrado conservation (Vieira *et al*. 2022).

In this context, the following hypotheses were evaluated: (i) Gall inducers, adapted to stressful habitats, enable *C. brasiliense* plants near drier *Veredas* to maintain similar gall diversity compared to those near wetter *Veredas*. (ii) Plants function as adaptive zones for galling insects, showing spatial patterns in gall distribution within individuals and populations to minimize interspecific competition for induction and development sites. (iii) *C. brasiliense* plants and galls near drier *Veredas* exhibit anatomical and histochemical attributes that enhance desiccation resistance compared to those near wetter *Veredas*. By comparing data from contrasting environments, we aim to better understand how functional tissue attributes vary in response to environmental changes. Additionally, we explore whether these attributes can serve as biological markers of local dryness and discuss their impacts on the resilience of these intricate biological systems.

## MATERIAL AND METHODS

### Study sites

The study was conducted at two sites within the Neotropical savannah (Cerrado *sensu stricto*), situated in the Peruaçu River Valley, northern Minas Gerais State. These sites are the *Parque Estadual Veredas do Peruaçu* (PEVP) in Cônego Marinho (14°56′13″ S, 44°37′44″ W) and the *Área de Proteção Ambiental do Rio Pandeiros* (APA Rio Pandeiros) in Bonito de Minas (15°21′37.2″ S, 44°54′45.9″ W). Both areas contain *Veredas* at varying stages of drying, with different water availability levels for local plant communities. At PEVP, the Peruaçu *Vereda* is in an advanced stage of drying, with groundwater levels ranging from 2 to 13 m below ground level, while the Almescla *Vereda* is beginning to dry out, with groundwater levels varying from 0 to 2 m below ground level (Nunes *et al*. 2022). These changes have significantly impacted the flora at Peruaçu the most, favouring species typical of the Cerrado *sensu stricto* in the open areas of the *Veredas*, formerly occupied by hygrophilous vegetation (Nunes *et al*. 2022). Both conservation units lie within an ecotone between the Cerrado and Caatinga biomes, featuring a mosaic of vegetation structures where palm swamps are common but Cerrado *sensu stricto* dominates (Fagundes *et al*. 2019). The climate, classified as tropical semi‐humid (Aw) according to the Köppen climate classification, exhibits distinct dry and rainy seasons, with an average annual rainfall of 1,000 mm and average temperatures ranging from 22°C to 24°C (Alvares *et al*. 2013). The study sites were sampled in May 2021. We chose to sample in May because it represents the end of the rainy season (October to March) and the beginning of the dry season (April to September). In this way, our analysis would not be influenced either by the heavy summer rainfall or by extreme dryness typical of the late winter season.

### Analysis of richness, abundance and co‐occurrence

At each study site, 20 individuals of *C. brasiliense* located along the margins of the *Veredas* were sampled. From each individual, five terminal branches were selected, ensuring that canopies were free from shading and other interference to standardize light exposure across all leaves and galls. Four compound leaves per terminal branch (totaling 400 leaves per site) were examined for various gall morphotypes to assess morphotype richness, abundance per individual, and co‐occurrence of morphospecies. To compare morphospecies richness and abundance between sites (Peruaçu and Almescla), the occurrence and total numbers of each morphospecies were recorded for 20 individuals per site. Parametric one‐way ANOVA tests were employed due to normal data distribution, confirmed by tests for variance homogeneity (Levene's test) and normality (Shapiro–Wilk test). To investigate competition hypotheses, probabilistic co‐occurrence analyses were conducted using the R package ‘co‐occur’ (Griffith *et al*. 2016). This analysis focused on species pairs, employing null models to compare observed and expected co‐occurrence patterns of gall‐inducing species per site, based on gall morphospecies presence/absence per host plant.

### Structural and histochemical analyses

Samples were collected from eight *C. brasiliense* individuals (four from each study site), consisting of non‐galled leaves (n = 8) and mature galls (n = 8 per morphospecies), including intralaminar globoid gall (IGG), extralaminar globoid gall (EGG), globoid petiolar gall (GPG), lenticular gall (LG), and clavate gall (CG) (*sensu* Isaias *et al*. 2013). The IGG, EGG, LG and CG are hymenopteran‐induced (Leite 2014), while the inducer of GPG remains unidentified. Mature galls were selected based on their size and anatomical features, including fully differentiated tissues, such as nutritive tissues, sclerenchymatous tissues, and common storage tissues. The samples were fixed in 50% FAA solution for 48 h, followed by dehydration in an ethanolic series according to Kraus & Arduin (1997). They were then embedded in Historesin (Leica Historesin Embedding Kit), cross‐sectioned at 5 μm using a rotary microtome (Leica 2035 Biocut), stained with 0.05% toluidine blue in 0.1 M phosphate buffer (pH 4.0) (O'Brien *et al*. 1964), and mounted in Acrilex colourless glass varnish (Paiva *et al*. 2006). Digital images were captured using a Leica DM500 microscope equipped with a Leica ICC50 digital camera and LAZ ES software, v. 1.8.1, from Leica Microsystems (Switzerland).

Histochemical tests were conducted using fresh and fixed samples of non‐galled leaves (n = 4) and galls (n = 4 per morphospecies) collected from both study sites (Peruaçu and Almescla). To detect reducing sugars, the Fehling's test was employed (Sass 1951) using equal parts of 6.93% copper II sulfate w/v and 34.6% potassium sodium tartrate with 12% sodium hydroxide w/w/v, heated to preboiling temperature; brown precipitates indicate positive results. Total lipids were visualized using 30% Sudan red III solution for 10 min at room temperature (Johansen 1940; Brundet *et al*. 1991); droplets stained orange–red indicate positive results. Total phenolics were detected with 1% ferric chloride solution (Johansen 1940) for 5 min; black precipitates indicate positive results. For lignin detection, Wiesner's reagent of acidified phloroglucinol was used (Johansen 1940); lignified cell walls stain light red.

### Histometric analyses

Digital images were analysed using the software AxioVision v. 4.8.2 (Zeiss Imaging Systems; Carl Zeiss Microscopy, LLC, White Plains, USA). We measured the thickness of the tissues composing the non‐galled leaves (adaxial and abaxial epidermis, palisade parenchyma and spongy parenchyma) and galls (adaxial and abaxial epidermis, common storage tissue, sclerenchymatous sheath, typical nutritive tissue and sclerenchymatous cells) (n = 4 galls per morphospecies from different individuals; five measurements per gall tissue) for both sites (Peruaçu and Almescla). The delimitation of gall tissues was based on Ferreira *et al*. (2019) (see Supporting Information—Figure S1). To compare the anatomical attributes between the study sites, the histometric data were analysed by *t*‐test (total leaf thickness) or two‐way ANOVA followed by Tukey's test, after being subjected to Levene's test for homogeneity of variances and the Shapiro–Wilk test of normality at 5% significance (*α* = 0.05). The thickness of different tissues was the response variable, and the explanatory variables were the study sites, the gall morphospecies, and the interaction between them. Statistical analyses were performed using base‐R/stats package in the R software (R Core Team 2021).

## RESULTS

### Abundance and co‐occurrence of galls

**Fig. 1:**
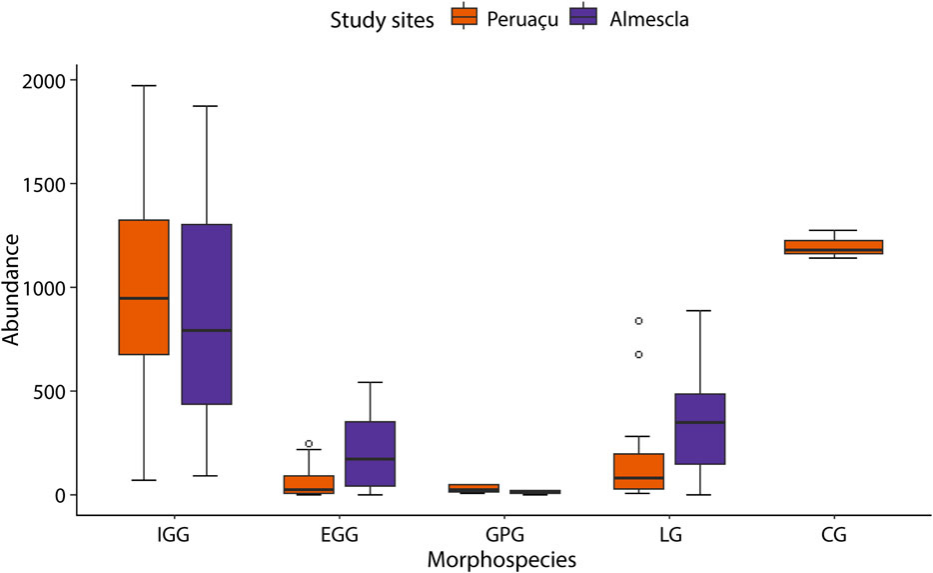
Abundance of five gall morphospecies on *Caryocar brasiliense* (Caryocaraceae) in the *Parque Estadual Veredas do Peruaçu* (PEVP) and the *Área de Proteção Ambiental do Rio Pandeiros* (APA Rio Pandeiros), with different groundwater levels. Note higher abundance of galls at APA Rio Pandeiros than at PEVP. (°) outliers. CG, clavate gall; EGG, extralaminar globoid gall; GPG, globoid petiolar gall; IGG, intralaminar globoid gall; LG, lenticular gall.

Samples amounted to 51,336 galls and five morphospecies were identified: IGG, EGG, GPG, LG, and CG. Among these, IGG, EGG, GPG and LG were found in both the Almescla and Peruaçu *Veredas*, while CG was exclusively observed in Peruaçu. Overall, Almescla exhibited a higher total abundance of galls compared to Peruaçu (Fig. 1). However, when analysing each morphospecies individually, IGG and GPG did not show significant differences between the sites (*P* = 0.925 and *P* = 0.377, respectively). In contrast, EGG and GPG were significantly more abundant in Almescla (*P* = 0.024 and *P* = 0.006, respectively). Co‐occurrence patterns varied between sites but were not statistically significant, suggesting random co‐occurrence of morphospecies pairs in both areas (*P* ≥ 0.05). IGG was notably present in nearly all co‐occurrence scenarios, indicating it is the most abundant morphospecies in both study sites.

### Morphoanatomical profiles of leaves and galls

**Fig. 2:**
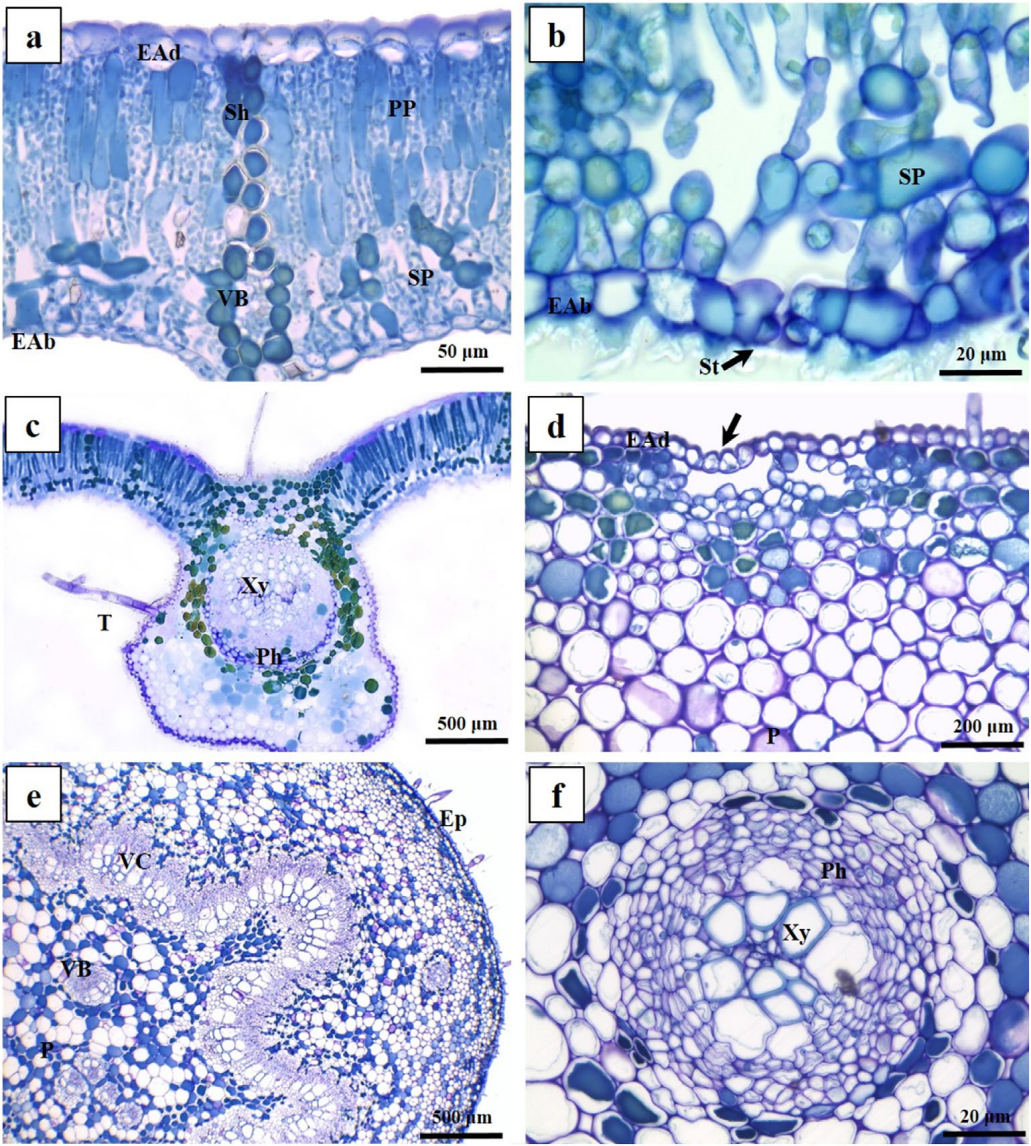
Leaf anatomy of *Caryocar brasiliense* (Caryocaraceae) in transverse section. (A) General organization of the mesophyll. (B) Detail of abaxial epidermis; stomata (arrow). (C) General organization of a second‐order vein. (D) Detail of petiole epidermis; stomatal crypt (arrow). (E) General organization of petiole. (F) Detail of amphicribal vascular bundle. EAb, epidermis on abaxial surface; EAd, epidermis on adaxial surface; Ep, epidermis; P, parenchyma; Ph, phloem; PP, palisade parenchyma; Sh, sheath; SP, spongy parenchyma; T, trichomes; VB, vascular bundle; VC, vascular cylinder; Xy, xylem.

*Caryocar brasiliense* exhibits coriaceous leaves featuring a uniseriate epidermis covered by a thick cuticle, with tector trichomes (Fig. 2A,C,D). The mesophyll is dorsiventral, consisting of two‐layered palisade parenchyma facing the adaxial surface, with inconspicuous intercellular spaces (Fig. 2A). The three‐layered spongy parenchyma facing the abaxial surface has rounded cells with thin sinuous primary walls and conspicuous intercellular spaces (Fig. 2A,B). Stomata are exclusively on the abaxial surface, always associated with prominent substomatal chambers (Fig. 2B). Veins are prominent, with collateral vascular bundles sheathed by sclerified cells extending to the adaxial epidermis (Fig. 2C). The petiole has a uniseriate epidermis covered by a thick cuticle and tector trichomes; crypts with stomata occur on the abaxial epidermis (Fig. 2D). The cortex consists of a three‐layered chlorophyllous parenchyma and several layers of angular collenchyma (Fig. 2D,E). The vascular system is arranged collaterally, forming a vascular cylinder with xylem and phloem surrounded by the innermost layer of the cortex (Fig. 2E). Accessory amphicribal bundles are observed in the medullary region of the vascular system and in the subepidermal cortical collenchyma (Fig. 2F). Leaves from both study sites (Peruaçu and Almescla) exhibit similar morphological and anatomical characteristics, indicating no qualitative differences.

**Fig. 3:**
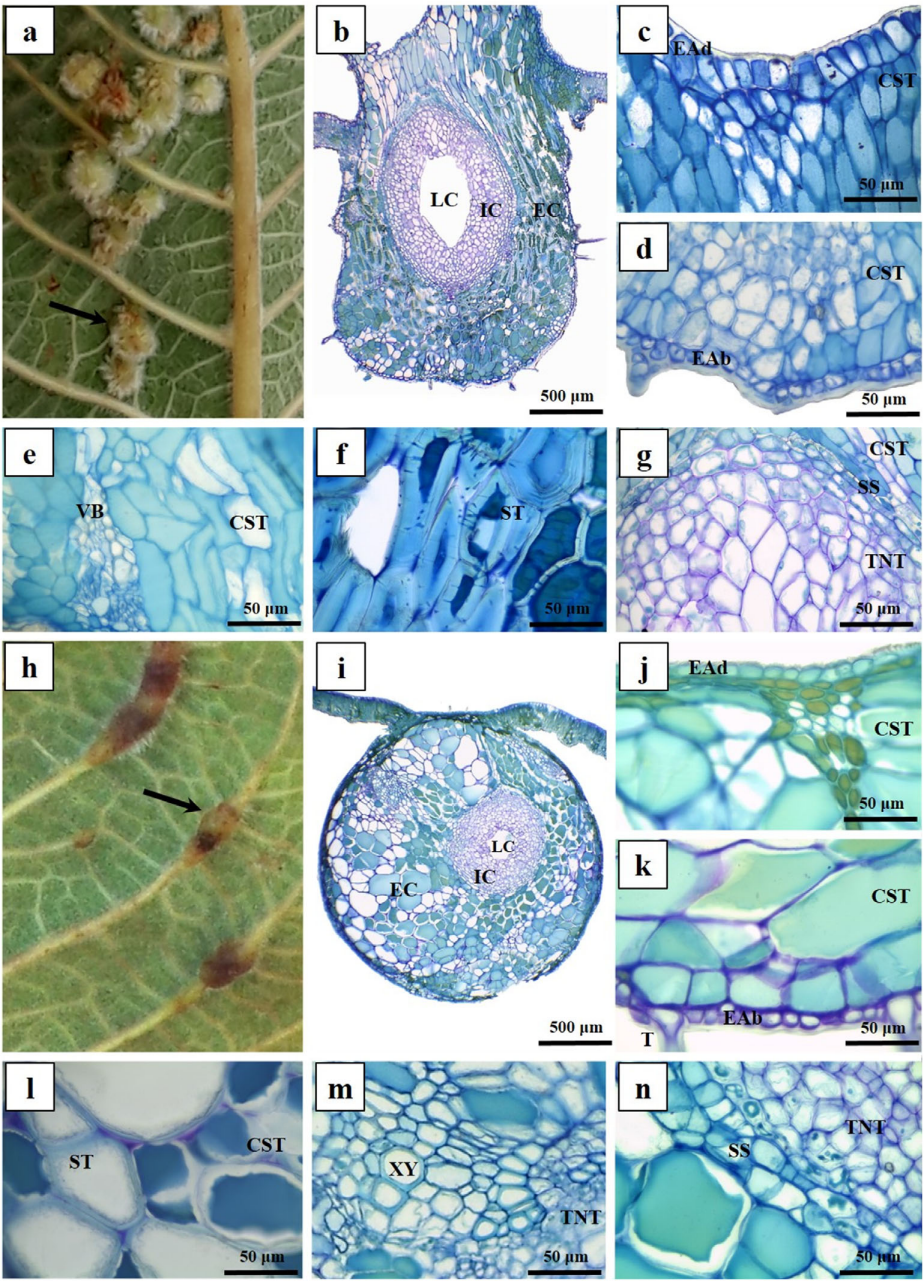
Morphoanatomical aspects of leaf galls on *Caryocar brasiliense* (Caryocaraceae). Transverse section, (A–G) Intralaminar globoid galls (IGG); (H–N) Extralaminar globoid galls (EGG). (A) Morphological aspects of IGG (arrow). (B) General organization, with internal and external tissue compartments. (C) Adaxial epidermis. (D) Abaxial epidermis. (E) Detail of common storage tissue and neoformed vascular bundles. (F) Detail of lignified parenchymatous cells; sclerenchymatous tissue. (G) Detail of sclerenchymatous sheath delimiting typical nutritive tissue. (H) Morphological aspects of EGG (arrow). (I) General organization, with internal and external tissue compartments. (J) Adaxial epidermis. (K) Abaxial epidermis. (L) Detail of lignified cells of common storage tissue; sclerenchymatous tissue. (M) Vascular bundles. (N) Detail of sclerenchymatous sheath delimiting typical nutritive tissue. CST, common storage tissue; EAb, epidermis on abaxial surface; EAd, epidermis on adaxial surface; EC, external tissue compartment; IC, internal tissue compartment; LC, laval chamber; SS, sclerenchymatous sheath; ST, sclerenchymatous tissue; T, trichomes; TNT, typical nutritive tissue; VB, vascular bundle; Xy, xylem.

The IGG morphospecies occurs in the leaf blade, often grouped or rarely isolated, being densely pilous and green coloured (Fig. 3A). It has two tissue compartments: external (EC) and internal (IC) (Fig. 3B). The EC is externally delimited by the uniseriate epidermis with thick cuticle and tector trichomes (Fig. 3C,D). The cells on the adaxial surface are columnar (Fig. 3C), while the cells of the abaxial surface are smaller and isodiametric (Fig. 3D). A parenchymatous common storage tissue is formed by several layers of anticlinally elongated cells facing the adaxial surface and isodiametric cells facing the abaxial surface (Fig. 3C–E). Neoformed vascular bundles occur laterally or abaxially in the common storage tissue (Fig. 3E). Common storage cells show lignified walls, forming a mechanical zone together with the sclerenchymatous sheath (Fig. 3F), which delimits the internal tissue compartment. The IC is formed by typical nutritive tissue and has 12–20 layers of small, isodiametric cells around the larval chamber (Fig. 3G).

The EGG morphospecies forms in the first‐ or second‐order veins, either isolated or coalescing, with yellowish green or light brown colour (Fig. 3H). It has two tissue compartments: external (EC) and internal (IC) (Fig. 3I); the EC is composed of uniseriate epidermis with isodiametric cells, thick cuticle and tector trichomes on both sides (Fig. 3J,K). The cortical collenchyma and parenchyma of the veins are transformed into the gall's common storage tissue, which has isodiametric lignified cells during maturation (Fig. 3L). The vascular system consists of vascular bundles, retained from the original vein structure (Fig. 3M). The IC is encased in a sclerenchymatous sheath and is characterized by typical nutritive tissue, comprising small, isodiametric to irregular‐shaped cells (Fig. 3N).

**Fig. 4:**
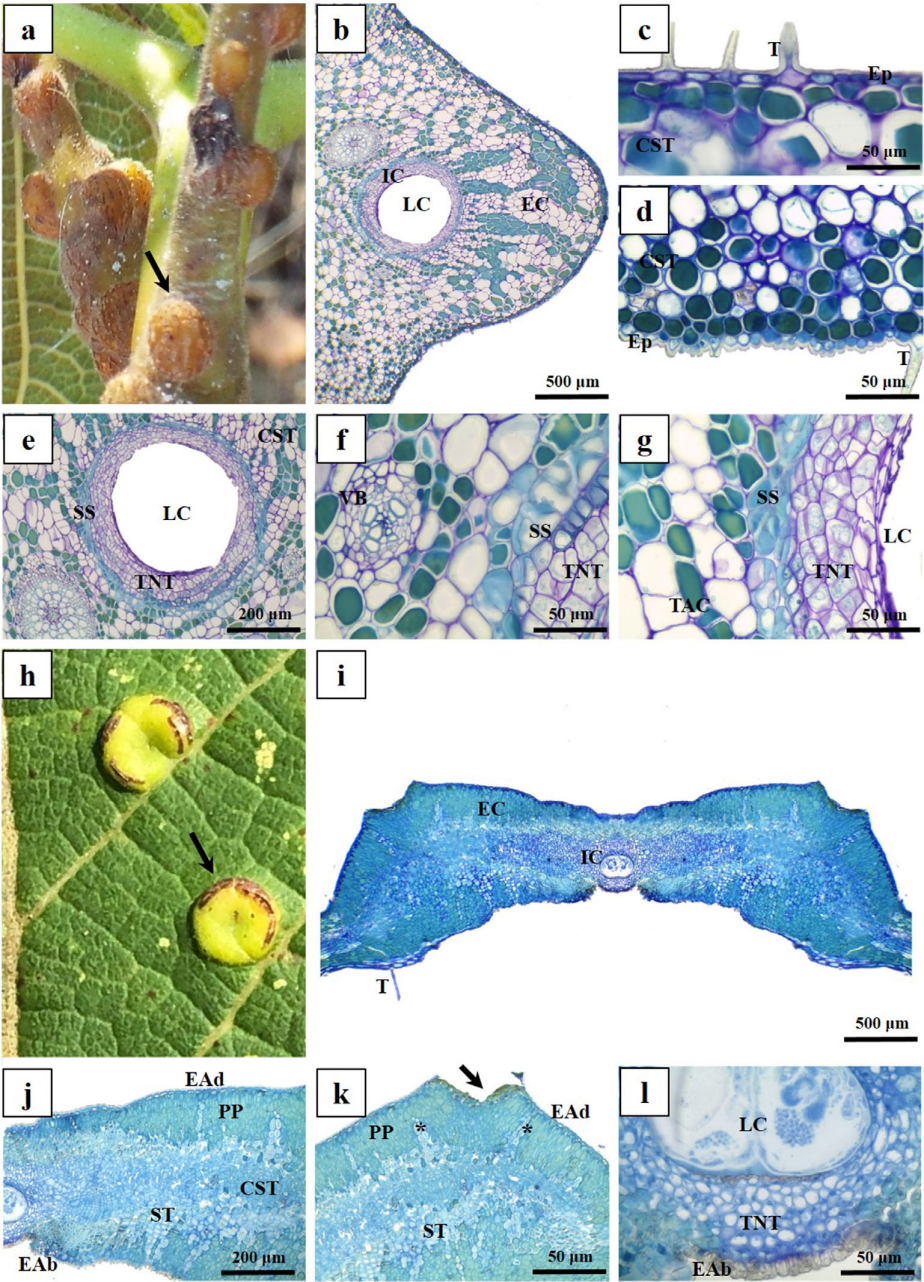
Morphoanatomical aspects of leaf galls on *Caryocar brasiliense* (Caryocaraceae). Transverse section, (A–G) Globoid petiolar gall (GPG); (H–M) Leticular gall (LG). (A) Morphological aspects of GPG (arrow). (B) General organization, with internal and external tissue compartments. (C) Adaxial epidermis. (D) Abaxial epidermis. (E) Detail of larval chamber surrounded by typical nutritive tissue, sclerenchymatous sheath, and common storage tissue. (F) Detail of concentric vascular bundle and sclerenchymatous sheath. (G) Detail of sclerenchymatous sheath and common storage tissue. (H) Morphological aspects of LG with suberous scars (arrow). (I) General organization, with internal and external tissue compartments. (J) Detail of the epidermis and common storage tissue with lignified cells; sclerenchymatous tissue. (K) Palisade parenchyma and epidermal injuries on adaxial surface (arrow); see remnants of bundle sheaths (asterisks). (L) Detail of typical nutritive tissue near abaxial surface. CST, common storage tissue; EAb, epidermis on abaxial surface; EAd, epidermis on adaxial surface; EC, external tissue compartment; Ep, Epidermis; IC, internal tissue compartment; LC, larval chamber; PP, palisade parenchyma; SS, sclerenchymatous sheath; ST, sclerenchymatous tissue; T, trichomes; TNT, typical nutritive tissue; VB, vascular bundle; Xy, xylem.

The GPG morphospecies is induced in the petiole cortex, either grouped or isolated, and has light green to light brown colour, like the petiole (Fig. 4A). It has two tissue compartments, external (EC) and internal (IC) (Fig. 4B), the EC being limited externally by uniseriate epidermis, with thick cuticle and trichomes on both sides. Epidermal cells are flattened or isodiametric on the abaxial or the abaxial surface, respectively (Fig. 4C,D). Subepidermal parenchyma differentiate into a common storage tissue with isodiametric cells (Fig. 4E). The vascular system is formed by vascular bundles of the petiole cortex that remain unchanged (Fig. 4F). The IC is delimited by a sclerenchymatous sheath surrounding the typical nutritive tissue, which features small, irregularly thickened‐walled cells of various shapes (Fig. 4G).

The LG morphospecies are induced in the leaf blade, project toward both leaf surfaces, and have light‐yellow colour (Fig. 4H). LG has two tissue compartments: the external (EC) and the internal (IC) (Fig. 4I). The EC is delimited by uniseriate epidermis with thick cuticle and tector trichomes on both sides. The chlorophyllous parenchyma of the leaf re‐differentiates into the common storage tissue of the gall, which is composed of palisade parenchymatous cells at the periphery, isodiametric cells toward the center, and a few layers of sclerenchymatous tissues that separate them from the internal compartment (Fig. 4J,K). In some regions, the epidermis is interrupted by suberous scars (Fig. 4K, arrow) and the remaining cells of the bundle sheath may be observed among the palisade cells of the common storage tissue (Fig. 4K, asterisks). The IC is formed by a typical nutritive tissue around the larval chamber, with small isodiametric cells delimited abaxially by a thick‐walled epidermis (Fig. 4L).

**Fig. 5:**
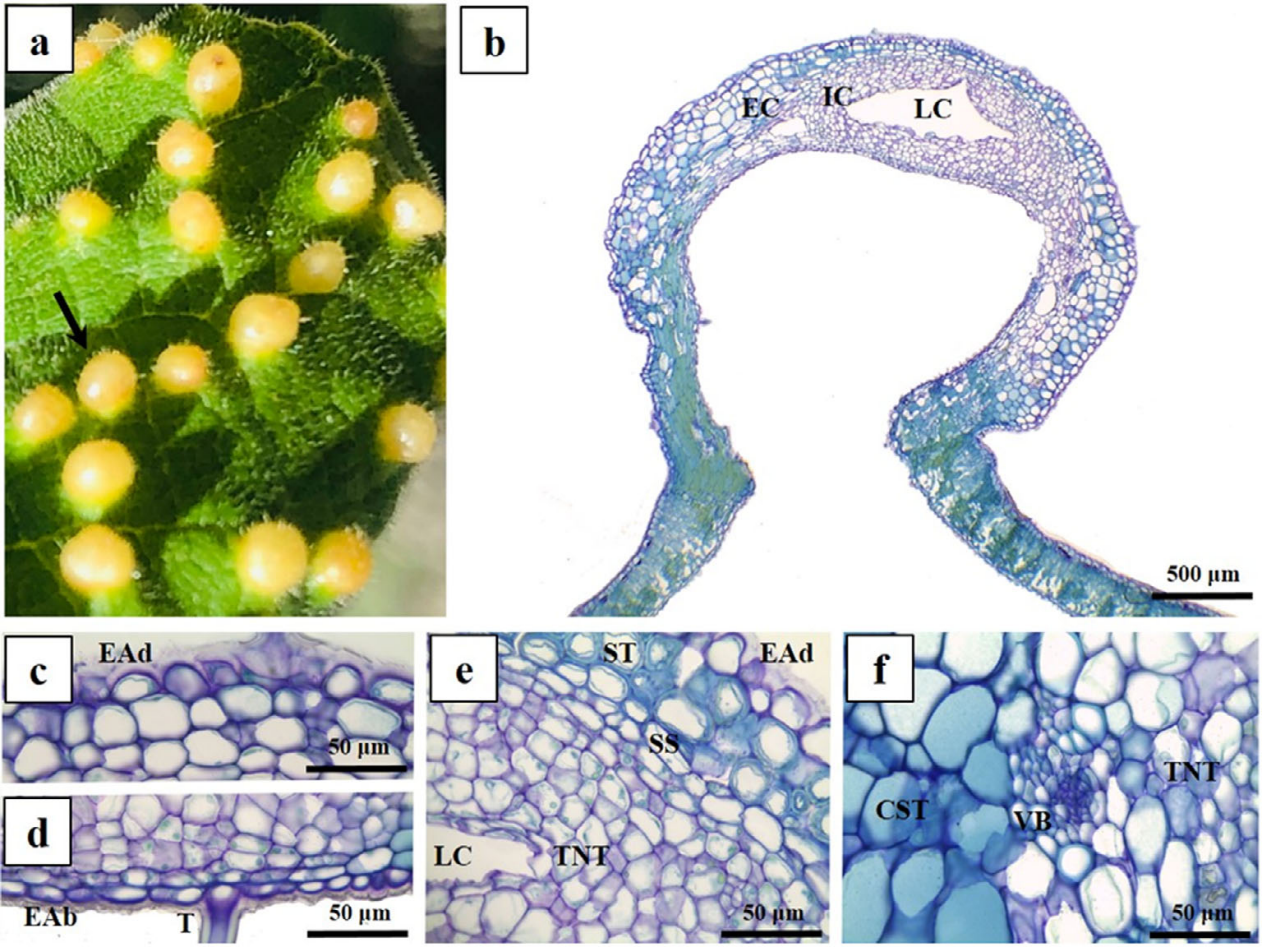
Morphoanatomical aspects of clavate leaf galls (CG) on *Caryocar brasiliense* (Caryocaraceae). Transverse sections. (A) Morphological aspects of CG (arrow). (B) General organization, with internal and external tissue compartments. (C) Adaxial epidermis with flanged cuticle. (D) Abaxial epidermis. (E) Detail of epidermis, sclerenchymatous tissue, sclerenchymatous sheath, and typical nutritive tissue. (F) Detail of neoformed collateral vascular bundle between common storage tissue and typical nutritive tissue. CST, common storage tissue; EAb, epidermis on abaxial surface; EAd, epidermis on adaxial surface; EC, external tissue compartment; IC, internal tissue compartment; LC, larval chamber; SS, sclerenchymatous sheath; ST, sclerenchymatous tissue; T, Trichomes; TNT, typical nutritive tissue; VB, vascular bundle.

Finally, the CG morphospecies is induced in the leaf blade; it is extra‐laminar, projects toward the adaxial surface, and is yellow‐orange in colour (Fig. 5A). It has two tissue compartments: the external (EC) and the internal (IC) (Fig. 5B). In the EC, the epidermis is uniseriate with a thick‐flanged cuticle and tector trichomes on both sides; epidermal cells are rounded in shape on the adaxial surface and more flattened on the abaxial surface (Fig. 5C,D). Parenchymatous cells re‐differentiate into the common storage tissue cells, with either isodiametric or periclinal elongated cells (Fig. 5C,E). Some cells of the common storage tissue are lignified, forming a mechanical zone (sclerenchymatous tissue) together with the sclerenchymatous sheath (Fig. 5E). The IC is composed of typical nutritive tissue with small irregularly‐sized cells, mostly isodiametric (Fig. 5E). Neoformed collateral vascular bundles are observed embedded in the sclerenchymatous sheath and in the typical nutritive tissue, close to the boundaries between the EC and the IC (Fig. 5F).

### Histometric analyses of leaf and gall structure

Histometric comparisons of *C. brasiliense* leaves between study sites revealed no differences in the total thickness (849.23 μm in Peruaçu vs. 857.99 μm in Almescla; *t* = 0.21292, *P* = 0.8325). Measurements of individual leaf tissues (adaxial epidermis, palisade parenchyma, spongy parenchyma and abaxial epidermis) were statistically similar between sites (Table 1).

**Table 1:** Results of two‐way ANOVA comparing tissue thickness of leaves and galls induced on *Caryocar brasiliense* (Caryocaraceae). Leaves are compared between study sites (Peruaçu and Almescla *Veredas*) and galls are compared between study sites and morphospecies (IGG, EGG, GPG, LG and CG).

tissue thickness (response variables)		explanatory variables	*F*‐value	*P*‐value
leaves	Adaxial epidermis	Study sites	705.824	0.993
	Abaxial epidermis		558.497	0.326
	Palisade parenchyma		452.756	0.816
	Spongy parenchyma		1230.991	0.129
galls	Abaxial epidermis	Study sites	35.087	0.051
		Morphospecies	3.850	<0.001
		Study sites × Morphospecies	0.576	0.632
	Adaxial epidermis	Study sites	103.970	0.037
		Morphospecies	4.430	<0.001
		Study sites × Morphospecies	0.569	0.636
	Common storage tissue	Study sites	302.797	<0.001
		Morphospecies	22.800	<0.001
		Study sites × Morphospecies	2.729	0.046
	Sclerenchymatous sheath	Study sites	492.725	<0.001
		Morphospecies	13.450	<0.001
		Study sites × Morphospecies	3.325	0.039
	Sclerenchymatous tissue	Study sites	342.693	0.001
		Morphospecies	10.890	<0.001
		Study sites × Morphospecies	36.230	<0.001
	Typical nutritive tissue	Study sites	209.260	<0.001
		Morphospecies	85.880	<0.001
		Study sites × Morphospecies	36.290	<0.001

Note: Variables expressed in mean thickness (μm) for each tissue layer. *P* ≤ 0.05 indicate significant differences.

Abbreviations: CG, clavate gall; EGG, extralaminar globoid gall; GPG, globoid petiolar gall; IGG, intralaminar globoid gall; LG, lenticular gall.

In galls, tissue thickness varied according to gall morphospecies, study site (Peruaçu and Almescla) and the interaction between them. Tissue thickness among gall morphospecies differed significantly for all tissues (Tables 1 and 2). The adaxial epidermis varied 123% among gall morphospecies, being IGG > CG > GPG > EGG > LG (Table 2). The abaxial epidermis varied 68%, being GPG > IGG > EGG > LG > CG (Table 2). The common storage tissue varied 575%, being thickest in GPG, followed by EGG, IGG, LG, and CG, which had the thinnest measurements (Table 2). The sclerenchymatous tissue was 243% thicker in EGG, followed by IGG, CG and LG, with the thinnest measures GPG not present this tissue (Table 2). The sclerenchymatous sheath varied 302% among gall morphotypes, being thickest in GPG, followed by EGG, IGG and CG (LG did not present this tissue) (Table 2). The typical nutritive tissue varied 257% between the thickest and thinnest gall morphospecies, being GPG > IGG > LG > EGG > CG (Table 2).

**Table 2:** Mean values of tissue thickness (μm) in both study sites (Peruaçu, Almescla and both) of each gall morphospecies induced on *Caryocar brasiliense* (Caryocaraceae).

morphospecies	study site	adaxial epidermis	abaxial epidermis	common storage tissue	sclerenchymatous tissue	sclerenchymatous sheath	typical nutritive tissue
IGG	Peruaçu	33.90 ± 4.69 a	22.00 ± 2.65 a	497.27 ± 125.82 a	342.15 ± 55.26 a	26.32 ± 5.10 a	118.25 ± 24.05 b
	Almescla	28.01 ± 4.88 b	21.70 ± 2.76 b	364.18 ± 52.80 b	267.73 ± 55.02 b	24.57 ± 5.70 b	268.46 ± 56.71 a
	Total	30.96 ± 5.40 A	21.85 ± 2.71 B	430.73 ± 117.21 C	304.94 ± 66.53 B	25.44 ± 5.48 C	193.35 ± 86.82 B
EGG	Peruaçu	21.00 ± 3.18 a	21.90 ± 3.42 a	548.21 ± 58.37 a	548.26 ± 89.80 a	29.87 ± 3.64 a	91.66 ± 18.38 b
	Almescla	18.50 ± 2.30 a	21.70 ± 2.90 b	526.48 ± 89.80 b	526.48 ± 58.37 b	29.59 ± 3.33 b	123.42 ± 35.37 a
	Total	19.75 ± 3.06 D	21.80 ± 3.17 C	537.35 ± 76.51 B	537.37 ± 76.51 A	29.73 ± 3.49 B	107.54 ± 32.35 D
GPG	Peruaçu	22.92 ± 1.91 a	23.40 ± 3.02 a	899.36 ± 112.19 a	n.a.	77.17 ± 7.59 a	301.59 ± 32.92 b
	Almescla	21.58 ± 1.81 b	22.10 ± 1.95 b	840.99 ± 110.65 b	n.a.	66.14 ± 11.18 b	313.16 ± 34.17 a
	Total	22.25 ± 1.98 C	22.75 ± 2.62 A	870.18 ± 115.18 A	n.a.	71.65 ± 11.03 A	307.37 ± 34.04 A
LG	Peruaçu	14.40 ± 2.30 a	19.90 ± 3.08 b	359.07 ± 52.33 a	163.12 ± 39.13 a	n.a.	138.77 ± 35.98 b
	Almescla	13.40 ± 1.96 a	20.30 ± 4.07 a	291.54 ± 95.40 b	150.05 ± 41.45 b	n.a.	156.49 ± 30.78 a
	Total	13.90 ± 2.20 E	20.10 ± 3.62 D	325.31 ± 84.02 D	156.58 ± 40.84 D	n.a.	147.63 ± 34.63 C
CG	Peruaçu	24.48 ± 6.44	13.55 ± 2.01	129.00 ± 24.71	175.38 ± 35.43	17.81 ± 3.92	85.99 ± 15.03
	Almescla	n.a.	n.a.	n.a.	n.a.	n.a.	n.a.
	Total	24.48 ± 6.44 B	13.55 ± 2.01 E	129.00 ± 24.71 E	175.38 ± 35.43 C	17.81 ± 3.92 D	85.99 ± 15.03 E

Note: n.a., not available either because of absence of tissue in the gall morphospecies (GPG and LG) or absence of gall morphospecies at study site (CG absent in Almescla). Different lowercase letters in columns indicate significant differences between sites for each of the gall morphospecies. Different uppercase letters in columns indicate significant differences among all gall morphospecies.

Abbreviations: CG, clavate gall; EGG, extralaminar globoid gall; GPG, globoid petiolar gall; IGG, intralaminar globoid gall; LG, lenticular gall.

When compared between study sites, it was observed that the adaxial and abaxial epidermis, sclerenchymatous tissue and sclerenchymatous sheath had greater thickness in the Peruaçu *Vereda*, the drier and most stressful environment (Tables 1 and 2). The adaxial epidermis was thicker by 21% for IGG, 14% for EGG, 6% for GPG and 7% for LG, while the abaxial epidermis was significantly thicker only for IGG and GPG (Table 2). The common storage tissue was 37% thicker for IGG, 4% for EGG, 7% for GPG and 23% for LG (Table 2); sclerenchymatous tissues were 28% thicker for IGG, 4% for EGG and 9% for LG (GPG did not present this tissue), while the sclerenchymatous sheath was 7% thicker for IGG, 1% for EGG and 17% for GPG (LG did not present this tissue) (Table 2). Unlike the other tissues, the typical nutritive tissue was significantly thicker in the Almescla *Vereda* (Tables 1 and 2), being 127% thicker for IGG, 35% for EGG, 24% for GPG and 13% for LG (Table 2). CG was not compared between sites because it was observed only in the Peruaçu *Vereda*.

The interaction between study sites and gall morphospecies evidence that these explanatory variables strongly influence the thickness of typical nutritive tissue and sclerenchymatous tissue, while the sclerenchymatous sheath and common storage tissue are relatively less influenced by this interaction. Epidermal tissues are not influenced by the interaction of study sites and morphospecies (Table 1).

### Histochemical analysis

Reducing sugars, lipids, lignins, and phenolic compounds were detected in both non‐galled leaves and galls from both sites (Table 3). In galls, reducing sugars were observed in the common storage tissue of IGG, GPG, LG and CG, as well as in the typical nutritive tissue of IGG, EGG, GPG and CG. Lipids were stained with Sudan III in the common storage tissue of IGG, GPG, LG and CG and more intensely in the typical nutritive tissue of IGG, EGG, GPG and CG (Table 3). Lignins and phenolic compounds were evidenced in the sclerenchymatous sheath of IGG, EGG, GPG, LG and CG and in sclerified parenchyma cells at the outer cortex of IGG, EGG, LG and CG. The histochemical profiles of galls from both sites were similar; however, lignins and phenolic compounds were more intensely stained in the IGG and EGG of the Peruaçu *Vereda*, with no differences observed for the other compounds (Table 3).

**Table 3:** Histolocalization of primary and secondary metabolites in galls induced on *Caryocar brasiliense* (Caryocaraceae) in different study sites (PEVP—Peruaçu *Vereda*, and APA Rio Pandeiros—Almescla *Vereda*).

histochemical reactions	outer tissue compartment										inner tissue compartment				
	common storage tissue					sclerenchymatous tissue					typical nutritive tissue				
	IGG	EGG	GPG	LG	CG	IGG	EGG	GPG	LG	CG	IGG	EGG	GPG	LG	CG
*Peruaçu*															
Reducing sugars	**+**	−	**+**	**+**	**+**	−	−	−	−	−	**+**	**+**	**+**	**−**	**+**
Lipids	**+**	−	**+**	**+**	**+**	**+**	**+**	**−**	**−**	**−**	**+**	**+**	**+**	−	**+**
Lignins	−	−	−	−	−	**++**	**++**	**+**	**+**	**+**	**−**	**−**	**−**	**−**	−
Phenolic compounds	**+**	**+**	**+**	**+**	**+**	**++**	**++**	−	**+**	**+**	−	−	−	−	−
*Almescla*															
Reducing sugars	**+**	−	**+**	**+**	n.a.	−	−	**+**	**+**	n.a.	**+**	**+**	**+**	−	n.a.
Lipids	**+**	−	**+**	**+**	n.a.	**+**	**+**	**+**	**+**	n.a.	**+**	**+**	**+**	−	n.a.
Lignins	−	−	−	−	n.a.	**+**	**+**	**+**	**+**	n.a.	**−**	**−**	**−**	**−**	n.a.
Phenolic compounds	**+**	**+**	**+**	**+**	n.a.	**+**	**+**	−	**+**	n.a.	−	−	−	−	n.a.

Note: n.a., not available because of absence of CG gall morphospecies in Almescla. Symbols: (−) negative reaction; (+) positive reaction.

Abbreviations: APA Rio Pandeiros, Área de Proteção Ambiental do Rio Pandeiros; CG, clavate gall; EGG, extralaminar globoid gall; GPG, globoid petiolar gall; IGG, intralaminar globoid gall; LG, lenticular gall; n.a., not evaluated; PEVP, Parque Estadual *Veredas* do Peruaçu.

## DISCUSSION

This study examined galls in drying natural environments by comparing traits from the community at the anatomical level, revealing significant impacts of human activities. Abundance and co‐occurrence analyses indicate that disturbed areas, despite being drier and more stressful, do not have more galls, contradicting classical hypotheses for gall distribution, such as the environmental stress hypothesis (Fernandes & Price 1988, 1992). The random co‐occurrence of galls suggests a lack of competition, as they occupy different leaf sites. This paper also examines the poorly explored topic of the anatomy of Hymenopteran‐induced galls from the neotropics, confirming classic patterns described in the literature for palearctic galls (Bronner 1992; Stone & Schönrogge 2003) and reporting new morphofunctional traits via histochemical analyses. It shows, for the first time, that gall morphogenesis, including its organization and functioning, not only depends on the gall‐inducing taxa but also responds to environmental conditions by increasing the thickness of tissue compartments under the stressful conditions of drying environments. Thus, gall structure is discussed as a potential bioindicator of environmental quality, particularly in response to increased dryness.

### Gall abundance, but not richness, is affected by dryness

The distribution and survival of gall‐inducing insect communities depend on environmental characteristics (Fernandes & Price 1992). Studies in the Cerrado indicate that environmental stressors increase gall‐inducing insect richness (Fernandes & Price 1992; Araújo 2017), which this study did not corroborate, as similar morphospecies richness were found at both sites. The Cerrado's seasonality and naturally stressful environment may mean that the stress levels in both areas were insufficiently different to affect the association of gall inducers to *Caryocar brasiliense*. As *C. brasiliense* is not typical of palm swamp environments, it does not rely on constant water supply from water‐saturated soils and copes with the Cerrado's dry conditions through desiccation defence mechanisms (Ramos *et al*. 2015), favouring the expression of adaptive anatomical traits in galls (Fagundes *et al*. 2018). These traits, which have been discussed in detail in the text, protect galling insects from environmental stress and explain how they are still able to associate with plants at the Peruaçu *Vereda*. However, our data show higher gall abundance in the less stressful Almescla *Vereda*, partially refuting the hypotheses that galls benefit from environmentally stressful habitats (Fernandes & Price 1988), including that of the uppermost crown portion of *C. brasiliense*, which is considered a harsh environment that allegedly benefits gall inducers (Leite *et al*. 2022a).

Understanding the plant–gall–environment relationship is complex and challenging, especially when water availability is considered, as it regulates the uptake of mineral nutrients that play fundamental physiological roles in the development of both plants and galls (Arriola *et al*. 2024). In the case of *C. brasiliense* and its galls, it has been shown that trees growing in clayey nutrient‐rich soils that hold more moisture shelter more galls than trees growing in well‐drained sandy and nutrient‐impoverished soils (Leite *et al*. 2022b). Thus, higher gall abundance in *C. brasiliense* near the Almescla *Vereda* is probably related to higher soil moisture, which ensures higher uptake of both water and nutrients, as predicted by Arriola *et al*. (2024). In turn, lower gall abundance, observed in Peruaçu, likely results from lower water and nutrient uptake, leading to lower survival, suggesting that gall inducers in the Cerrado are sensitive to environmental changes due to drying processes. Thus, conservation efforts should focus on understanding how ongoing climate changes and low water availability may affect gall biodiversity. For example, the CG morphospecies is restricted to the degraded Peruaçu *Vereda* and may be at risk of local extinction before being fully studied. Further analyses on their phenology and on the dispersal capacity of their inducers are needed to understand why CG only occurs at the Peruaçu *Vereda*, where it was relatively more abundant than other gall morphotypes, and how it deals with a stressful drying environment despite having relatively thinner tissue layers.

Beyond the effects of the environment on galls, the role of competition in structuring galling insect communities has been widely studied (Fagundes *et al*. 2018, 2020; Ramos *et al*. 2019). These studies suggest that null processes in communities vary between environments, with co‐occurrence in xeric environments and biotic drivers influencing community structure (Ramos *et al*. 2019), which was not corroborated herein. Fagundes *et al*. (2020) argued that interspecific competition shapes galling insect communities through the selection of gall induction sites and development stages, allowing different taxa to coexist on the same host plant and avoid competition. In fact, Leite *et al*. (2022a) showed that galls associated with *C. brasiliense* are equally distributed in the different parts of the tree crowns. This is confirmed by our results, as all analysed galls occupied different induction microsites—leaf blade, veins and petiole—representing distinct anatomical resources for gall development and favouring the occurrence of different gall morphotypes within the same organ.

### General aspects of gall structure

Galls induced in *C. brasiliense* display structural and metabolic traits consistent with literature patterns (Bronner 1992; Ferreira *et al*. 2019), including typical nutritive tissue and internal compartment for nutrient storage. Galls vary in complexity: those induced by hemipterans and other sucking insects are simpler and parenchymatous, while hymenopteran‐ and dipteran‐induced galls are complex, with specialized tissues (Mani 1964; Stone & Schönrogge 2003; Ferreira *et al*. 2019), like those studied herein. Leite (2014) attributed the induction of IGG to a species of *Eurytoma* sp. (Hymenoptera: Eurytomidae), EGG was assigned to *Bruchophagus* sp. (Hymenoptera: Eurytomidae), CG to an undescribed species of the family Eulophidae (Hymenoptera), and LG to an undescribed species of Hymenoptera. Despite Hymenoptera being a major gall‐inducing order worldwide (Oliveira *et al*. 2016), few Neotropical studies focus on the morphoanatomical and histochemical patterns of their galls. So far, in addition to those described here, only the galls induced by *Plastobelyta gallicola* Kieffer and *Espinosa nothofagi* Grahan (Pteromalidae) (Aguilera *et al*. 2022) and *Eurytoma* sp. (Eurytomidae) (Castro *et al*. 2012) have been described anatomically. The patterns observed here, such as concentric specialized tissue layers, for example, nutritive tissues, sclerenchymatous sheath, and cortical parenchyma, align with existing literature (Castro *et al*. 2012; Isaias *et al*. 2018; Ferreira *et al*. 2019; Aguilera *et al*. 2022), reinforcing current paradigms on gall anatomy. An exception seems to be the case of the globoid galls induced by *P. gallicola* (Pteromalidae) on *Nothofagus obliqua* (Mirb.) Oerst (Nothofagaceae), found in Chile, that are mostly parenchymatous and structurally simple (Aguilera *et al*. 2022), indicating that the anatomical patterns of hymenopteran galls are not absolute.

Regardless of gall complexity, tissue hyperplasia and cell hypertrophy are common in galls induced by different taxa, leading to ground tissue homogenization (Rohfritsch 1992; Isaias *et al*. 2014; Ferreira *et al*. 2019). Although parenchyma homogenization is a common feature, gall development and functional tissue compartmentalization are directed by gall inducers (Bronner 1992; Oliveira *et al*. 2016), thus influencing the generation of different morphotypes (Isaias *et al*. 2014), or morphospecies (Teixeira *et al*. 2022). In fact, histometric comparisons of the same tissue lineages among gall morphospecies were all significantly different, reinforcing that galls are extended phenotypes of their inducers (Stone & Schönrogge 2003) at both tissue and cell levels (Carneiro & Isaias 2015). Further evidence of gall complexity relies on vascularization patterns (Mani 1964; Bronner 1992; Bragança *et al*. 2022), as observed in all hymenopteran‐induced galls on *C. brasiliense*, either by the maintenance of the pre‐existing vascular bundles or by the neoformation of new ones. Vascular tissues are generally less plastic, as they usually retain their original functions of transporting water and nutrients, thus being the less altered tissues in galls (Isaias *et al*. 2014); the fact that galls maintained the structural patterns of their host organs (amphicribal in the petioles, where GPG occurs, and collateral in the leaf lamina, where all the others occur) reinforces such reduced plasticity. In fact, vascular tissues may function as a ‘plant constraint’ for the development of galls, especially in stem galls (Jorge *et al*. 2022), but also in galls on other plant organs, since the functionality of vascular tissues—transport of water, nutrients, and other molecules—is rarely altered in galls. An exception to that rule appears to be the case of LG, in which no vascular bundles were detected. This suggest that, in this gall, even the vascular bundles undergo redifferentiation and lose their function, which, to the best of our knowledge, has not previously been reported in specialized literature on the anatomy of galls. In that case, the delivery of water and nutrients to gall developmental sites must occur via symplastic and/or apoplastic cell–cell flux from nearby vascularized non‐galled tissues toward the gall, as hypothesized to occur in other galls for the delivery of water and nutrients (Arriola *et al*. 2020; Ferreira *et al*. 2020). Although *C. brasiliense* galls are induced by hymenopterans and have similar characteristics, significant structural–functional differences were found between some of them, for example, reducing sugars were evidenced in the common storage tissue, unlike the starch and secondary metabolites reported for the storage tissues in the classical literature (Bronner 1992; Rohfritsch 1992). This highlights the need for more studies on galling Hymenoptera in the Neotropics to understand their impact on the functionalities of the host plant tissues and organs.

### Structural and metabolic markers of gall responses to the environment

The anatomy of *C. brasiliense* leaves shows xeromorphic characteristics, such as a thick cuticle, abundant palisade parenchyma, few intercellular spaces, and lignified cells around the vascular bundles (Fahn & Cutler 1992; Bieras & Sajo 2009), which are adaptive anatomical traits of plants subjected to stressful dry environments. Among the xeromorphic characteristics of plants, lignified cells make the leaves more rigid and long‐lived, reducing water loss and allowing the recovery of turgor after periods of water scarcity (Oguchi *et al*. 2003). In this sense, we used anatomical attributes such as tissue thickness, indumentum density and variations in the deposition of secondary walls as structural stress markers in galls, as well as the accumulation of defence‐related secondary metabolites (e.g. polyphenols, lignins) as metabolic stress markers. In galls, these markers can be observed in functionally distinct tissue compartments: the internal compartment, related to the nutrition of gall inducers, and the external compartment, whose functionality is related to their protection against external factors, whether biotic or abiotic (Carneiro *et al*. 2014;Bragança *et al*. 2017; Ferreira *et al*. 2019). Although the adaptive nature of gall morphology, including anatomy, has been extensively discussed in literature (see Mani 1964; Stone & Schönrogge 2003; Isaias *et al*. 2014, 2024; Ferreira *et al*. 2019), deviations from the expected anatomical organization, especially the overexpression of the stress markers mentioned above, are important to understand how galls cope with drying processes that drastically change the natural environments.

In the dermal system, the thick cuticle and/or presence of periderm are characteristics that attenuate water stress, corroborating the microenvironmental hypothesis (Stone & Schönrogge 2003; Castro *et al*. 2012) by providing further hygrothermal protection to the galls. In the ground tissues, lignified cells and tissues forming a mechanical zone are considered a protection against natural enemies commonly reported for galls occurring in different environments (Oliveira *et al*. 2006; Ferreira *et al*. 2019). However, lignification processes may favour the maintenance of physiological processes in plant tissues in the face of water restriction, as already reported for the increase in leaf resistance under xeric conditions (Lima *et al*. 2018). In the case of *C. brasiliense* galls, the lignified parenchyma cells and the sclerenchymatous sheath in the external compartment confer mechanical resistance against the effects of desiccation and the attacks of natural enemies, as commonly discussed in the literature (Carneiro *et al*. 2014; Isaias *et al*. 2014; Carneiro & Isaias 2015). Also, these tissues provide the galls with an energy supply and chemical protection, as they are composed of living cells that accumulate both primary and secondary metabolites, thus being an active part of the gall's common storage tissue (*sensu* Ferreira *et al*. 2019). In fact, the tissues of the external compartment, such as the epidermis and the common storage tissues (parenchyma, lignified parenchyma and sclerenchymatous sheath), were thicker for the galls from the Peruaçu *Vereda*, a drier environment, which corroborates the hypothesis that the external compartments of galls play active roles in protection against external factors (Bragança *et al*. 2017; Ferreira *et al*. 2019). Similar anatomical acclimation responses have been reported to occur in galls subjected to both sun and shade conditions (Castro *et al*. 2023); however anatomical responses of galls to degraded drying environments are herein reported for the first time.

The fact that gall tissues acclimate to the environment shows that, as is true for any other plant organ, the phenomenon of phenotypic plasticity is indeed ubiquitous in plants (Palacio‐López *et al*. 2015) and that galls are in fact true plant organs (Ferreira *et al*. 2019), often very distinct from the non‐galled plant organs. This is a particularly interesting topic to highlight in the association of *C. brasiliense* and its galls because the non‐galled leaves from both sites are anatomically and histometrically similar, but their galls are not. This is most probably related to phenological aspects of both leaves and galls; while *C. brasiliense* leaves are produced during the rainy season (Vilela *et al*. 2008) under conditions of high‐water availability, galls start to develop only after the peak of the rainy season, when leaves are already expanding. Seasonality has been reported to affect gall morphogenesis, as the abundance of resources during the rainy season positively affects the weight, volume, and surface area of Cecidomyiidae galls induced on *Myrcia neoobscura* E.Lucas & C.E.Wilson (Myrtaceae) (Campos *et al*. 2023), but the anatomical aspects underlying such changes remain to be studied. In the case of *C. brasiliense* and its galls, however, anatomical data indicate that the morphogenesis of galls is sufficiently sensitive to express signs of resource limitations, in this case, water scarcity due to the end of the wet season and lower levels of the groundwater in the drier *Vereda*. This is especially important when considering that most of the water‐absorbing root system of *C. brasiliense* is usually distributed in superficial strata of the soil (up to 1.25 m below ground level) (Lima *et al*. 2015), and that the groundwater at the Peruaçu *Vereda* varies from 2 to 13 m below ground level (Nunes *et al*. 2022), thus subjecting plants and galls to a more stressful habitat when compared to the wetter *Vereda*, as is elegantly demonstrated by the histometric data.

In addition to the plasticity expressed by the external compartments of galls, the increased thickness of internal compartments of galls from the Almescla *Vereda*, an environment with more water availability, indicates that environmental integrity may positively affect the nutrition of galling insects inside their galls by promoting the differentiation of additional layers of the nutritive tissue. As mentioned above, although gall morphospecies are the variables that influence gall structure the most (Teixeira *et al*. 2022), the environment is shown here to play a crucial role on how galls allocate their resources, either to the external compartment, for protection, or to the internal compartment, for nutrition. This is somewhat related to the ‘dilemma of plants—to grow or to defend’ (Herms & Mattson 1992), in which the recruitment of secondary metabolites to play defensive roles (e.g. against natural enemies or, in this case, against oxidative stress) may affect some ontogenetic processes. As secondary metabolites integrate plant metabolism to undertake regulatory functions beyond the defensive ones (Erb & Kliebenstein, 2020), as previously discussed for galls (Isaias *et al*. 2014, 2015, 2024; Kuster *et al*. 2020), they most certainly have implications for the ecology of galls and of their inducers in the context of plant and gall morphogenesis subjected to degraded, drying habitats. For instance, IGG had the greatest histometric changes between the analysed environments, thus being phenotypically more plastic. Therefore, IGG is endowed with greater adaptability, which possibly explains its greater abundance in all scenarios when compared to the other gall morphospecies associated with *C. brasiliense*. GPG, however, was not the most abundant gall morphospecies, but like IGG, its abundance did not vary between Peruaçu and Almescla *Veredas*. While IGG is the most plastic gall, GPG has the thickest tissues in almost all scenarios, which indicates that increased tissue thickness, by itself, guarantees a suitable microenvironment for the development of gall inducers, despite the harsh surrounding environments, as much discussed in the literature (Mani 1964; Stone & Schönrogge 2003; Ferreira *et al*. 2019; Isaias *et al*. 2024).

The consequences of the dynamic balance of tissue thickness on the survival and on the fitness of galling herbivores and plants subjected to drier conditions should be the focus of future investigations. For instance, some gall inducers may be affected negatively by increased dryness, which seems to be the case for EGG and LG, whose abundances were lower in Peruaçu. Unlike IGG and GPG, EGG had the lowest tissue plasticity, and LG had the thinnest adaxial epidermis and lacks the sclerenchymatous sheath, an important protective tissue. It is worth mentioning that *C. brasiliense* galls have several natural enemies (Leite 2014; Leite *et al*. 2022a, 2022b) and their anatomical features seem less favourable in the face of enemy‐rich, drier and hotter environments in the Peruaçu *Vereda*. Thus, the effects of changing environments on the morphogenesis of galls should continue to be investigated to examine whether the trends observed herein also apply to other ecologically relevant systems.

Together with structural analyses, the histochemical profiles further support the roles played by the internal gall compartments, which are mostly associated with the nutrition of the gall inducer (Isaias *et al*. 2014; Ferreira *et al*. 2019), while the external compartments are linked to protective mechanisms, whether against desiccation or natural enemies (Cornell, 1983; Ferreira *et al*. 2019). In this study, nutritious substances, such as reducing sugars and lipids, were observed in the internal compartment, confirming what is already known for Hymenoptera‐induced galls (Bronner 1992; Isaias *et al*. 2018). This is also the case of lignin deposition and phenolics accumulation in the external compartment, which corroborates discussions on the adaptive strategies of gall maintenance and protection (Isaias *et al*. 2018; Ferreira *et al*. 2019). Herein, beyond this classical approach, such features are also understood as stress markers. Special attention should be paid to the sclerenchymatous tissues, which are classically described as being composed of dead cells (Fahn & Cutler 1992) but in galls are frequently demonstrated to be live, functioning, cells (Oliveira *et al*. 2010; Carneiro *et al*. 2014; Isaias *et al*. 2024). As mentioned before, the sclerenchymatous tissues in the galls on *C. brasiliense* are an active part of the external compartment, which responds by thickening under the drier environment of Peruaçu, but also have their histochemical profiles changed most between the study sites, with more intense labeling of phenolics and lignins in the galls at Peruaçu, the drier *Vereda*. Our hypothesis is that the accumulation of lignins and phenolics in the galls of *C. brasiliense* function as a chemical strategy to promote gall homeostasis by the scavenging reactive oxygen species, as previously hypothesized and discussed for other galls subjected to stressful conditions (Isaias *et al*. 2015; Ferreira *et al*. 2018; Guedes *et al*. 2022). According to this rationale, when under stress, the more lignified and phenolic‐rich the galls, the more stressful is the environment. Thus, lignin deposition and phenolics accumulation are stress markers in galls that reflect the severe degradation of the *Veredas* and not only affects the hygrophilous communities, but also the adjacent Cerrado *sensu stricto*, as demonstrated for the galls on *C. brasiliense*.

**Fig. 6:**
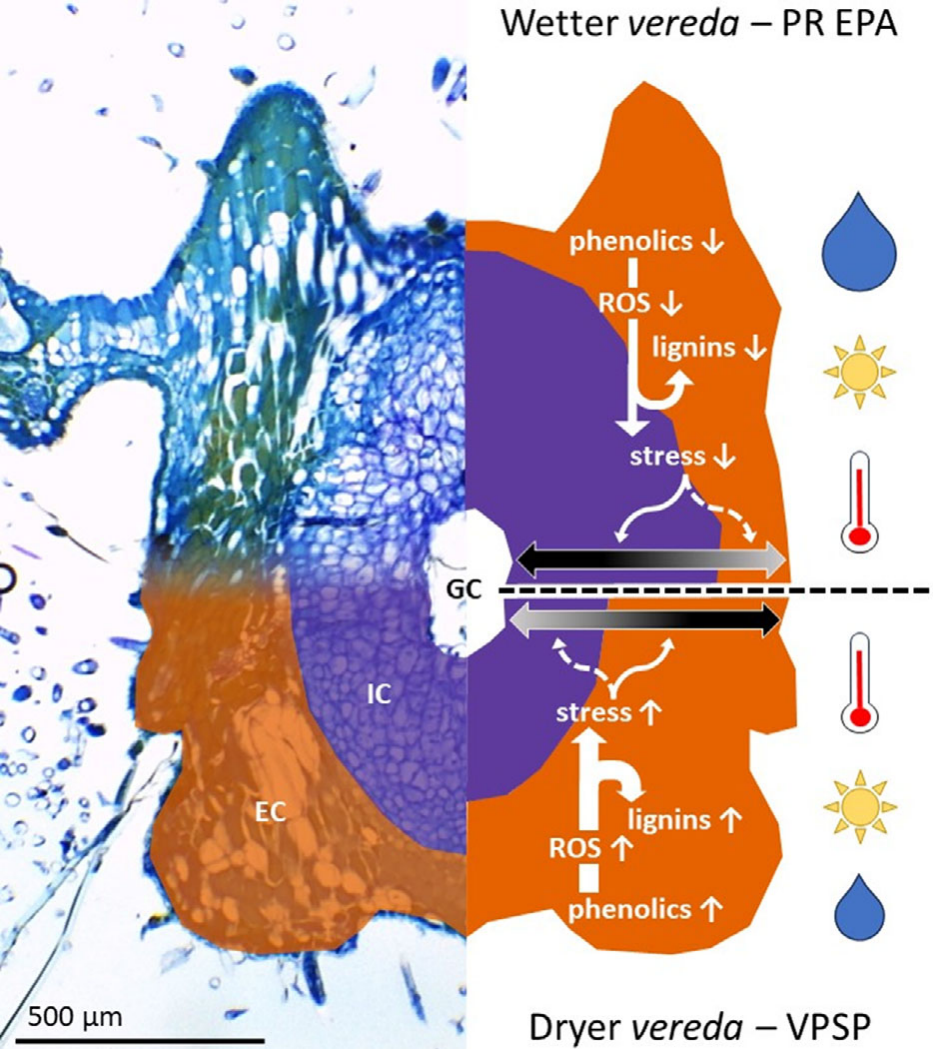
Schematic representation of mechanisms determining differential investment between internal (IC) and external (EC) tissue compartments of galls, based on empirical evidence and theoretical background (Isaias *et al*. 2015). On left: histological micrograph of the intralaminar globoid gall (IGG) showing IC (purple) and EC (orange). On right: contrasting conditions in wetter and drier *Veredas*, based on groundwater levels (Nunes *et al*. 2022). The balance of reactive oxygen species (ROS), phenolics and lignins affects stress at the gall tissue level, determining investment in IC or EC (double‐headed arrows with black–grey gradient indicate high–low investment). Upper right: wetter Almescla *Vereda* at the *Área de Proteção Ambiental do Rio Pandeiros*, where increased water supply leads to lower ROS and phenolics levels, resulting in lower lignin deposition. Less stress favours investment in IC over EC. Bottom left: drier Peruaçu *Vereda* at the *Parque Estadual Veredas do Peruaçu*, where reduced water supply leads to higher ROS and phenolics levels, resulting in higher lignin deposition. More stress favours investment in EC over IC.

In summary, the hypothesis‐driven comparison of different areas demonstrates that the distribution of galling insect communities does not always reflect environmental characteristics, such as local dryness, although these factors do affect gall abundance. The absence of co‐occurrence among gall morphospecies indicates that there is no competition among them, further corroborating that the plant body is composed of adaptive zones that constitute different niches for the establishment of gall‐inducers. Additionally, it was found that such galls share similar anatomical and metabolic characteristics, such as common storage tissue, typical nutritive tissue, sclerenchymatous cells and nutritious substances present in the internal tissue compartment. As far as the external tissue compartment is concerned, we found that the gall phenotype is influenced by the environment, expressing structural and histochemical characteristics commonly associated with the tolerance of plants to stressful conditions such as dryness. An example of this is the sclerenchyma, which confers mechanical–structural reinforcement to protect the innermost gall tissues, preventing cellular collapse and water loss to the external environment and favouring the tolerance of the gall‐inducing agents to water restriction. Under conditions of a wetter environment, galls were shown to produce larger internal compartments, whose functionalities are directed to nutrition of the gall inducer, suggesting that the allocation of resources for gall development is subject to a trade‐off mechanism between its compartments, widely influenced by external factors (Fig. 6). In this sense, anatomical and histochemical profiles evidence that galls do, in fact, express structural–functional attributes that can be understood as environmental stress markers in response to drying environmental processes.

## AUTHOR CONTRIBUTIONS

ISF, WSA, and RGSC conceptualized the study, designed the sampling protocol and contributed to manuscript preparation. ISF conducted the sampling and processing of the plant material.

## Supporting information


**Figure S1.** Schematic representation of gall tissues based on Ferreira *et al*. 2019 and of measurements taken during histometric analyses. (A) General view of the organization of gall tissues, with typical nutritive tissue (TNT) around the gall chamber (GC), followed outwards by the sclerenchymatous sheath (SS; may not be present in all galls), by the common storage tissue (CST), by the sclerenchymatous tissue (ST; may not be present in all galls), and by the epidermis (Ep; may be adaxial, if on top, or abaxial, if underneath the gall). (B) Detail of measurements taken for histometric analyses, in which the TNT was measured from the cells around the GC until the beginning of SS. SS was measured from the end of TNT until the beginning of CST. CST was measured from the end of SS until the beginning of the Ep (or, ST, if present). ST was measured from the end of CST and the beginning of the Ep. Ep was measured from the inner periclinal epidermal cell wall until the outer periclinal cell wall, including the cuticle.
