# Supplementary figures and images for: Gall responses to drying habitats: Insights from the community of galling herbivores associated with the superhost Caryocar brasiliense Cambess. (Caryocaraceae)
Source: Plant Biol (Stuttg). 2025 Apr 4;28(3):924–38. doi: 10.1111/plb.70008 (PMC13089609; doi:10.1111/plb.70008)

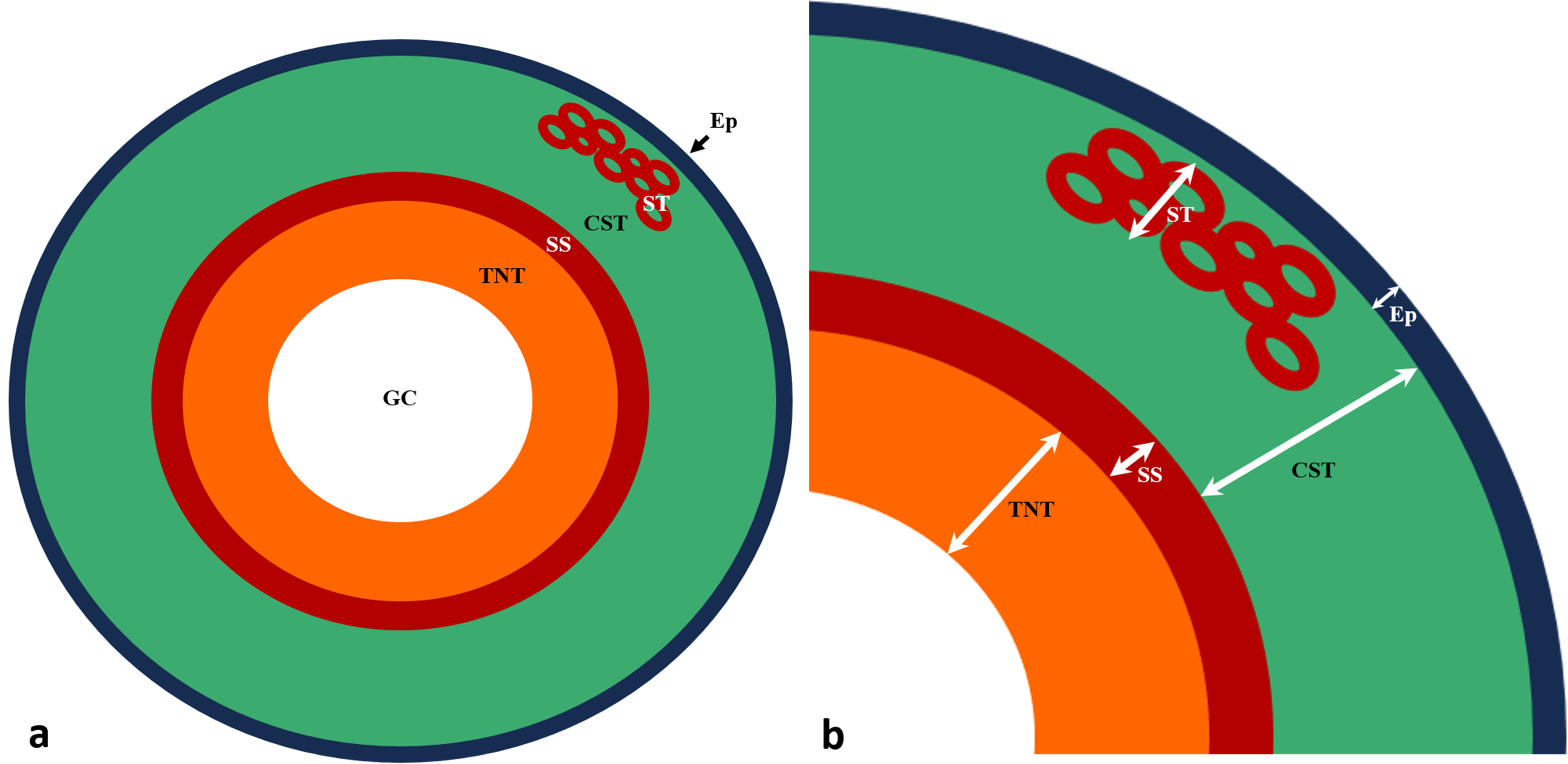

Supplement: Supplementary file 1 — Figure S1. Schematic representation of gall tissues based on Ferreira et al. 2019 and of measurements taken during histometric analyses. (A) General view of the organization of gall tissues, with typical nutritive tissue (TNT) around the gall chamber (GC), followed outwards by the sclerenchymatous sheath (SS; may not be present in all galls), by the common storage tissue (CST), by the sclerenchymatous tissue (ST; may not be present in all galls), and by the epidermis (Ep; may be adaxial, if on top, or abaxial, if underneath the gall). (B) Detail of measurements taken for histometric analyses, in which the TNT was measured from the cells around the GC until the beginning of SS. SS was measured from the end of TNT until the beginning of CST. CST was measured from the end of SS until the beginning of the Ep (or, ST, if present). ST was measured from the end of CST and the beginning of the Ep. Ep was measured from the inner periclinal epidermal cell wall until the outer periclinal cell wall, including the cuticle. [file PLB-28-924-s001.tif]
